# *Burkholderia pseudomallei* infection presenting with a lung abscess and osteomyelitis in an adult man: A case report

**DOI:** 10.1097/MD.0000000000012145

**Published:** 2018-08-21

**Authors:** Lei Huang, Zhi Yang, Xiao-Ping Zhou, Jun-Rong Wu

**Affiliations:** a Department of Clinical Laboratory; b Department of Nuclear Medicine, The Affiliated Tumor Hospital of Guangxi Medical University; c Department of Clinical Laboratory, The First Affiliated Hospital of Guangxi University of Chinese Medicine, Nanning, Guangxi, China.

**Keywords:** *burkholderia pseudomallei*, diagnosis, lung abscess, osteomyelitis

## Abstract

**Rationale:**

The frequency of infections caused by Burkholderia pseudomallei is increasing worldwide. Here, we report a case of B pseudomallei infection presenting with a lung abscess and osteomyelitis in an adult man.

**Patient concerns:**

A 38-year-old man presented with high-grade fever, productive cough, and chronic joint pain of the limbs. Examinations revealed multiple nodules, soft tissue mass shadows, pulmonary cavitation in bilateral lungs, and fluid and soft tissue swelling in bilateral hips.

**Diagnoses:**

The microbiologic diagnosis based on a positive culture revealed the etiologic agent to be B pseudomallei.

**Interventions:**

The patient was treated with intravenous ceftazidime and levofloxacin, and out-of-hospital treatment continued with oral cotrimoxazole.

**Outcomes:**

The patient responded well to the treatment.

**Lessons:**

subsections Because of its increasing incidence, B pseudomallei infection should be highly suspected among employees who work in laboratories and healthcare facilities. Misdiagnosis can lead to treatment failure and high mortality rates, especially among individuals working in laboratories in non-endemic areas; therefore, early and accurate diagnosis of B pseudomallei infection is essential. Adequate antimicrobial treatment and long-term follow-up are imperative to reduce morbidity and mortality.

## 1 Introduction

Melioidosis is an anthropozoonosis caused by *Burkholderia pseudomallei*, a non-fermenting gram-negative, facultative soil saprophyte. It is endemic in South-east Asia and Northern Australia and parts of South and Central America, an increasing number of emerging infectious diseases caused by *B pseudomallei* have been reported in many countries.^[1]^ The clinical presentation of the infection varies greatly, ranging from mild localized infection to acute fulminant sepsis with widespread bacterial dissemination. The lungs, subcutaneous tissue, musculoskeletal region, liver, and spleen are the organs predominantly affected.^[2]^ Although acute pulmonary infection is the most frequent form of the infection, acute septicemic infection with a high mortality rate is the most severe form of the infection. Osteomyelitis is described as the infection and destruction of bones and is mainly caused by aerobic or anaerobic mycobacterium and fungus; however, osteomyelitis of bilateral hips caused by *B pseudomallei* is rare. Here, we report a case of *B pseudomallei* infection presenting as a lung abscess and complicated osteomyelitis of bilateral hips in an adult man.

## 2 Case report

### 2.1 Patient information

A 38-year-old man was referred to our hospital for joint pain of the limbs for >4 months, fever for 10 days, and cough for 2 weeks. Before the current admission, he was treated with methylprednisolone tablets, tongfengding capsules, and lansoprazole tablets; however, his symptoms did not improve over time. He had no history of travel to any melioidosis epidemic areas or was not exposed to any animals. He was employed as an agricultural worker and had a history of heavy alcohol consumption and excessive smoking.

### 2.2 Clinical findings

On physical examination, the patient was toxic with high-grade fever (39.5°C), blood pressure of 121/65 mmHg, pulse rate of 106 per minute and respiratory rate of 21 per minute. In addition, he reported having >4 months of the bilateral shoulder, elbow, wrist, knuckle, hip, knee, ankle and left sternoclavicular pain that aggravated while performing daily activities. This limited his mobility and caused an inability to walk.

Blood test results revealed a total leucocyte count of 13.7 × 10^9^/L, including 92.3% neutrophils and 4.6% lymphocytes, platelet count of 841 × 10^9^/L, and hemoglobin level of 68 g/L. The levels of fasting C-reactive protein (CRP) and procalcitonin (PCT) were 165.60 mg/L and 1.03 ng/ml, respectively. Erythrocyte sedimentation rate (ESR) was 143 mm/h, and rheumatoid factor (RF) was 30.30 IU/mL, which were markedly elevated compared with normal levels (ESR: 0–15 mm/h and RF: 0.0–20.0 IU/mL, respectively). Serum total bilirubin was 128.1 (1.00–28.0) μmol/L, total protein was 40.7 (65.0–85.0) g/L, albumin was 22.0 (40.0–55.0) g/L, alanine aminotransferase was 154 (9–50) U/L, aspartate transaminase was 214 (15–40) U/L, alkaline phosphatase was 269 (40–150) U/L, γ-glutamyl transferase was 1782 (10–60) U/L. The serum urea, creatinine, and uric acid levels were within the normal levels. *Mycoplasma pneumoniae* antibodies (both IgM and IgG) were detected using the Diagnostic Kit for Measurement of Antibodies to *M pneumoniae* (Passive Particle Agglutination) (FUJIREBIO INC, Tokyo Japan), and the mixed antibody titer was 1:160, indicating that the patient was likely infected with *M pneumoniae* . The chemiluminescence microparticle immunoassay was used to detect human immunodeficiency virus (HIV) antigen (p24)/antibody (HIV-1/HIV-2) combo and the *Treponema pallidum* antibody. The immune colloidal gold method was used to detect IgG antibodies against *Mycobacterium tuberculosis*. All of the test results were negative.

### 2.3 Diagnostic assessment

Chest contrast-enhanced computerized tomography (CECT) revealed multiple nodules, soft tissue mass shadows, and pulmonary cavitation in the lower zone of bilateral lungs; thus, a pulmonary abscess was considered. CECT of the hip joint showed the destruction of bone of bilateral acetabulum, femoral head, and proximal femur, and interruption of bone in the right femoral neck. Nuclear magnetic resonance imaging (MRI) revealed fluid and soft tissue swelling in bilateral hips. Therefore, infectious osteomyelitis was considered.

The staining results of direct smears prepared from sputum and joint fluid from the hips for mycobacteria and fungi were negative. The blood culture using the BD BACTEC^TM^ FX (Bactec-Becton, Dickinson) was positive. Sputum, joint fluid, and blood were subsequently submitted for routine cultures. Small colonies were cultured on the blood plate for 1 day. After 2 days, the colony with hemi-hemolytic had grown into a medium size, was gray-yellow, and was shaped like a wheel or chrysanthemum. Pinkish rugose colonies were observed on MacConkey's agar. The organism with turbid growth, and then formed a ruffled bacteria membrane in liquid culture. All the growth colonies had a strong foul musty odor. The 2 ends of the bacteria were concentrated with gram-negative bacteria and had multiple flagella in the single extreme. The results of conventional biochemical testing revealed that the organism could decompose glucose, lactose, maltose, mannitol, levo-ribose, and sucrose by producing acid but not gas. It was not able to decompose left wood. Additionally, the tests showed that the organism was positive for phosphatase, oxidase, nitrate reductase, and had a negative reaction when tested for urea, tellurite, acetamide, citrate, malonate, esculin, lysine, tryptophan, and DNase. A microorganism identification test was conducted using the Microflex LT/SH system (Bruker Daltonics) and the results suggested that the organism was *B pseudomallei* as was suspected. Antibiotic susceptibility testing using the BD Phoenix 100 system showed that the organism was sensitive to trimethoprim/sulfamethoxazole, ticarcillin/clavulanic acid, piperacillin/tazobactam, piperacillin, levofloxacin, meropenem, ciprofloxacin, co-trimoxazole, ceftazidime, and imipenem and resistant to amikacin, aztreonam, tobramycin, and gentamicin.

### 2.4 Therapeutic intervention

On the basis of the culture results and the antibiotic susceptibility testing, the patient received 2 g of intravenous ceftazidime every 8 hours and 0.3 g of intravenous levofloxacin every 12 hours, in conjunction with proper nutrition therapy.

### 2.5 Follow-up and outcomes

**Figure 1:**
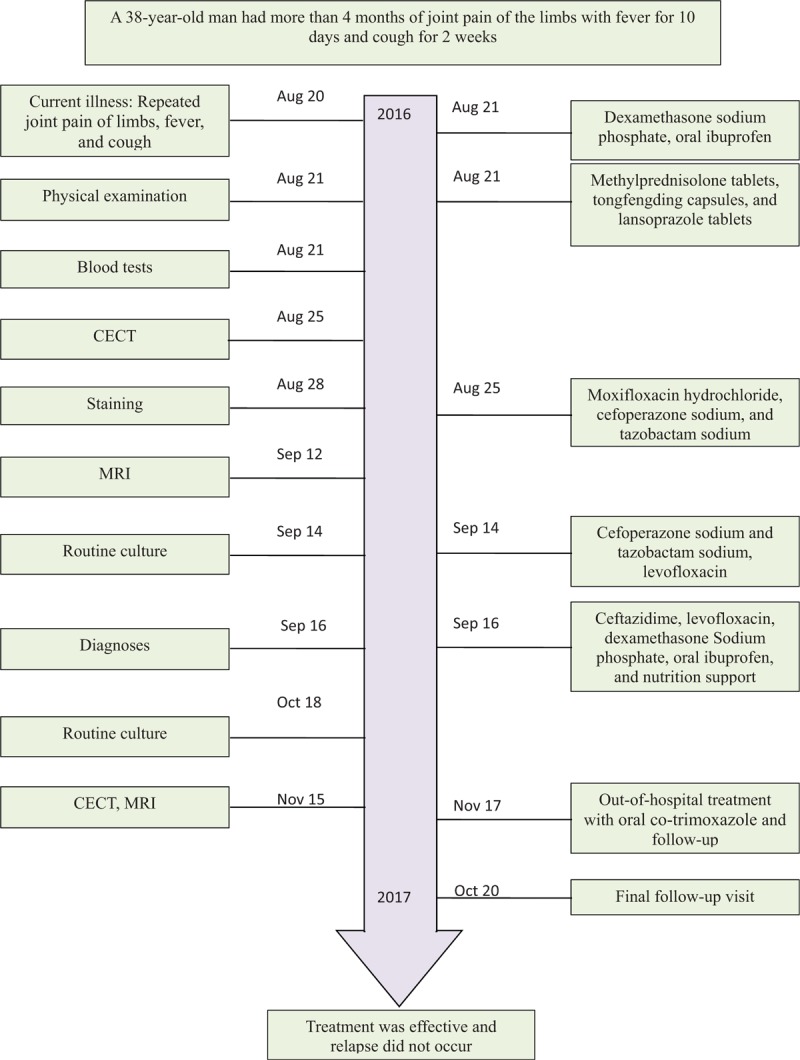
Timeline displaying the patient's diagnosis, treatment, and follow-up. CECT = contrast-enhanced computerized tomography, MRI = magnetic resonance imaging.

His fever and cough resolved after 7 days. The sputum culture was negative after 4 weeks and the blood culture was negative after 7 weeks. Chest CECT showed remarkable absorption of the pulmonary abscess after 2 months; however, there was still a small number of effusions in bilateral hips observed using MRI, and *B pseudomallei* was still isolated from the joint effusions. After 2 days of hospitalization, discharge plans were made as per the patient's request; the patient did not develop any complications during treatment in the hospital. Out-of-hospital treatment continued with 2 960 mg tablets of oral co-trimoxazole every 12 hourly for another 6 months and he was seen for follow-up visits once a month. At the 6-month follow-up visit, his joint effusions had disappeared without relapse. The diagnosis and treatment information for this case are summarized in Figure 1.

### 2.6 Ethical consideration

This study was prospectively performed and approved by the Institutional Ethics Committees of the First Affiliated Hospital of Guangxi University of Chinese Medicine and conducted according to the ethical guidelines of the Declaration of Helsinki. The patient gave written informed consent for publication.

## 3 Discussion

*B pseudomallei* is an environmental gram-negative bacillus that was first described by Whitmore and Krishnaswami in 1912^[3]^; it was predominately endemic in Southeast Asia in the 1990s. It commonly presents in soil and surface water of infected areas and can infect humans through entry through open cuts or wounds. The patient presented here was a farmer whose occupation was working on the farmland. This might have been the source of his melioidosis, however, ingestion, inhalation, and laboratory-acquired infection^[4]^ can also cause melioidosis. With the enhanced mobility of the global population, an increasing number of people with *B pseudomallei* infection have been reported from other parts of the world. In China, patients with *B pseudomallei* infection were mainly from the southern coastal region of China, for example, Hannan, Fujian, and Guangdong^[5,6]^; however, cases of infectious osteomyelitis of bilateral hips caused by *B pseudomallei*, as described in this report, are uncommon and rarely reported.

Risk factors for acquiring *B pseudomallei* infection include thalassemia, soil/water exposure, diabetes mellitus, excessive alcohol consumption, chronic lung disease, renal disease, systemic lupus erythematosus, and being of the male sex.^[7]^ The patient in our report consumed large amounts of alcohol and smoked cigarettes heavily. He was previously diagnosed with alcoholic liver disease, community-acquired pneumonia, and gouty arthritis, which significantly increased his risk of *B pseudomallei* infection. The incubation period for *B pseudomallei* infection is known to last for a few days up to several years.^[8]^ In this patient, the time from onset of symptoms to diagnosis was approximately 4.5 months. The clinical presentation of the disease varies greatly; it is often asymptomatic or presents with only mild fever and cough during the early period. The current patient was a farmer with a history of heavy smoking and drinking; thus, he probably did not realize that he was infected by a pathogen. He did not live in a *B pseudomallei* endemic area; there were no previous cases in which locals were infected with this disease in his area. Therefore, the patient likely did not seek timely treatment because of the lack of information and public awareness about the disease. *B pseudomallei* was recently recognized as an emerging infection in India; a study showed that the majority of patients were males from rural areas, however, the majority of cases remain to be diagnosed and reported.^[9]^ In countries where melioidosis is likely to occur, education on sanitation is needed in order to enhance knowledge on the epidemiology of disease in the countryside, to ensure that people fully understand the dangers and harms associated with the disease. If one suspects infection with *B pseudomallei*, it is recommended that medical treatment be sought in a timely manner and that doctor's instructions are adhered to. With increasing reports of this disease, we believe that *B pseudomallei* should be considered, particularly among farmers who are at higher risk of *B pseudomallei* infection. If sick farmers on standard flu medications do not see an improvement in health over time, microbiologic identification should be conducted to test for *B pseudomallei* infection. If diagnosed with *B pseudomallei* infection, adequate antimicrobial treatment and long-term follow-up are imperative to reduce morbidity and mortality.

Patients with increased serum levels of CRP and PCT should be highly suspected of bacterial infection. However, CRP levels are not a consistent marker of the severity of infection. PCT is a better diagnostic marker of general bacterial infections and it is very useful in distinguishing bacterial infections from non-bacterial infections. The serum PCT level of the patient was markedly elevated (1.03 ng/mL) compared with the normal level (0.00–0.05 ng/mL), indicating that he was more likely to be infected with bacteria. In such cases, blood cultures should be performed as quickly as possible as the most severe form of *B pseudomallei* is acute septicemic infection, which is accompanied by a high mortality rate. The current patient was diagnosed with bacteremia based on the results of the blood culture.

Although the clinical presentation of the disease varies greatly with acute, chronic, or subacute forms, an increasing number of *B pseudomallei* infections have been reported globally, as diagnosing *B pseudomallei* has become common. The “gold standard” method for the diagnosis of *B pseudomallei* is still the isolation of *B pseudomallei* from clinical specimens; culture-based methods have a low diagnostic sensitivity in patients with melioidosis. A high index of suspicion is needed both among employees in laborites and healthcare facilities as phenotypic differences in *B pseudomallei* are not easily recognized and they might be misinterpreted as *Burkholderia cepacia*,^[10]^ especially in laboratories in non-endemic areas. It takes approximately 1 week to confirm species using conventional biochemical tests; traditional biochemical tests and rapid automatic identification systems often experience errors in identification. Molecular tests for the rapid confirmation and differentiation of *B pseudomallei* have been widely used;^[11,12]^ although the sequencing of the 16S ribosomal RNA (rRNA) gene is widely used for the identification of this organism,^[13]^ it cannot confidently separate *B pseudomallei* and closely related species such as *Burkholderia thailandensis*. Commercial identification systems also have the same problems.^[14]^ The *groEL* gene sequences offer a higher discriminatory power between the 2 species than the 16S rRNA.^[15]^ Molecular detection is a reliable method to identify the bacteria but requires special equipment, which is not available in some microbiology laboratories, particularly in non-endemic areas, and diagnostic problems encountered in culture-negative cases remain largely unresolved. The sensitivities and specificities of serological tests for diagnosis remain to be evaluated.^[12]^ Immediate and accurate identification of *B pseudomallei* is essential as treatment of these infections require intensive and prolonged treatment.^[16]^

The results of antibiotic susceptibility testing in our case revealed that the organism was sensitive to the majority of antibiotics, which is similar to the findings from previous studies.^[12,17]^ Acute pulmonary infection is the most frequent form of the infection; thus, it is essential that sputum cultures are conducted when the presenting symptoms do not improve after “conventional therapy” (chloramphenicol, trimethoprim, and doxycycline), and that blood cultures are performed if necessary. Many clinical trials have reported the efficacy of various antimicrobial regimens for *B pseudomallei*.^[16,18–20]^ When *B pseudomallei* infection is diagnosed, intravenous antimicrobial therapy is initiated and should be continued for 10 to 14 days; thereafter 3 to 6 months of oral maintenance therapy should be provided to patients until abscesses are completely absorbed.^[2]^ Patients should be seen for a follow-up visit at least 6 months after the complete resolution of any abscess.

Treatment of *B pseudomallei* infections have been described in a number of previous studies; *B pseudomallei* infections were reversible or effectively controlled in some cases, however, some patients died because they were not able to access diagnosis and treatment. The immunity of our patient did not significantly decrease; the organism was sensitive to most antibiotics, particularly ceftazidime and imipenem, which is one of the primary reasons and antibacterial treatment was effective. Ceftazidime has been considered to be effective in the treatment of *B pseudomallei* infection.^[21]^ Simpson et al reported that ceftazidime and imipenem had the similar efficacy for the treatment of acute severe *B pseudomallei*, but more frequent treatment failure was observed in patients treated with ceftazidime.^[22]^ This was the first case of *B pseudomallei* diagnosed and treated in our hospital. Ceftazidime, given in combination with levofloxacin and out-of-hospital treatment that was continued with oral co-trimoxazole, seemed to be efficacious in the treatment of our patient. A previous case of *B pseudomallei* infection in a patient with diabetes was reported in Hunan province,^[23]^ but the bacteria were resistant to imipenem and was considered a carbapenem producing strain causing ineffective treatment with many antibiotic drugs. Furthermore, a bacterial culture was not performed when the patient first visited hospital with left arm suppurative infection, therefore, failure to appropriately diagnose and treat the patient with antibiotic therapy, likely lead to the death of this patient. Out-of-hospital treatment remains an important modality to reduce morbidity and mortality; Huffam et al suggested that out-of-hospital management of melioidosis with 24 hourly infusions of ceftazidime through a peripherally inserted central catheter line was safe and effective.^[24]^ Based on the findings from our case, a significant antibacterial effect was observed when adequate antimicrobial treatment, including ceftazidime and levofloxacin, was administered as quickly as possible, and out-of-hospital treatment was continued with oral co-trimoxazole therapy. In similar cases, we recommend that such therapy, including out-of-hospital treatment, be continued until the bacteria isolated from all materials is negative. The relapse rates of *B pseudomallei* are high^[25]^; thus, it is highly important to complete therapy and engage in long-term follow-up to avoid recurrence.^[26]^ We did not identify any relapse during the regular clinical and biologic monitoring visits our patient attending in the year proceeding treatment.

This case report was subject to several limitations. First, although this patient had some risk factors for *B pseudomallei* infection, we do not know exactly how he became infected. Second, the adverse effects or unanticipated events associated with treatment were not recorded. Third, follow-up was interrupted 1 year after discharge because of the inability to contact the patient; therefore, the long-term outcome and prognosis are unknown. Fourth, this case shows that ceftazidime plus levofloxacin were feasible and effective in the treatment of *B pseudomallei* infection; whether this treatment strategy has the same efficacy for other patients with *B pseudomallei* infection needs to be confirmed in future studies. Although this is the first case of *B pseudomallei* infection reported in our hospital, the patient received timely and accurate diagnosis and treatment, which makes us more confident regarding the future diagnosis and treatment of this disease. Additionally, we were able to collate relevant information and summarize our experience in the diagnosis and treatment of this rare case. We believe that our experiences in the management of this case will be useful for the diagnosis and treatment of *B pseudomallei* infection in future, in both our hospital and those in other settings.

## Author contributions

**Conceptualization:** Jun-rong Wu.

**Data curation:** Lei Huang.

**Formal analysis:** Xiao-Ping Zhou.

**Resources:** Lei Huang, Zhi Yang.

**Software:** Zhi Yang.

**Writing – original draft:** Xiao-Ping Zhou.

**Writing – review & editing:** Jun-rong Wu.
